# Hospital costs and prognosis in end-stage renal disease patients receiving coronary artery bypass grafting

**DOI:** 10.1186/s12882-020-01972-w

**Published:** 2020-08-08

**Authors:** Kuang-Ming Liao, Lu-Ting Kuo, Hsueh-Yi Lu

**Affiliations:** 1 Department of Internal Medicine, Chi Mei Medical Center, Chiali, Taiwan; 2 Division of Neurosurgery, Department of Surgery, National Taiwan University Hospital, Taipei, Taiwan; 3 Department of Industrial Engineering and Management, National Yunlin University of Science and Technology, Yunlin, Taiwan

**Keywords:** Coronary artery bypass grafting, End-stage renal disease, Hospital costs, Prognosis

## Abstract

**Background:**

Coronary artery disease is common in patients with end-stage renal disease (ESRD). Patients with ESRD are a high-risk group for cardiac surgery and have increased morbidity and mortality. Most studies comparing ESRD patients receiving coronary artery bypass grafting (CABG) or percutaneous coronary intervention have found that the long-term survival is good in ESRD patients after CABG. The aim of our study was to compare ESRD patients who underwent CABG with the general population who underwent CABG, in terms of prognosis and hospital costs.

**Methods:**

This study analyzed data from the National Health Insurance Research Database in Taiwan for patients who were diagnosed with ESRD and received CABG (ICD-9-CM codes 585 or 586) between January 1, 2004, and December 31, 2009. The ESRD patients included in this study all received catastrophic illness cards with the major illness listed as ESRD from the Ministry of Health and Welfare in Taiwan.

The control subjects were randomly selected patients without ESRD after propensity score matching with ESRD patients according to age, gender, and comorbidities at a 2:1 ratio from the same dataset.

**Results:**

A total of 48 ESRD patients received CABG, and their mean age was 62.04 ± 10.04 years. Of these patients, 29.2% were aged ≥70 years, and 66.7% were male. ESRD patients had marginally higher intensive care unit (ICU) stays (11.06 vs 7.24 days) and significantly higher ICU costs (28,750 vs 17,990 New Taiwan Dollars (NTD)) than non-ESRD patients. Similarly, ESRD patients had significantly higher surgical costs (565,200 vs. 421,890 NTD), a higher perioperative mortality proportion (10.4% vs 2.1%) and a higher postoperative mortality proportion (33.3% vs 11.5%) than non-ESRD patients.

**Conclusions:**

After CABG, ESRD patients had a higher risk of mortality than non-ESRD patients, and ICU and surgery costs were also higher among the ESRD patients than among patients without ESRD.

## Background

Cardiovascular disease is one of the most important diseases and accounts for most mortality in end-stage renal disease (ESRD) patients [1]. A previous study [2] showed that ESRD patients have a poorer prognosis than chronic kidney disease (CKD) patients without hemodialysis after coronary revascularization. In their study, coronary artery bypass graft (CABG) yielded better clinical outcomes in ESRD patients than percutaneous coronary intervention (PCI) did. Most studies have focused on comparing CABG versus PCI outcomes in ESRD patients [3–7].

Yoshiyuki et al. enrolled 152 ESRD patients requiring hemodialysis who underwent CABG and found that the long-term outcomes were not satisfactory and that factors of diabetes and peripheral artery disease were predictive of poor prognosis [8]. In 2004, Powell et al. [9] compared the prognosis of ESRD patients and patients without ESRD after CABG and showed that intraoperative and postoperative morbidity and mortality were not increased in ESRD patients. However, ESRD patients on hemodialysis who received CABG had longer hospital stays than patients without ESRD. There have been limited studies investigating the prognosis, risk factors and hospital costs of CABG between ESRD and non-ESRD patients. Thus, the aim of our study was to assess whether ESRD patients on hemodialysis who received CABG experienced higher hospital costs and perioperative and postoperative mortality than CABG patients without ESRD and to evaluate the factors related to outcomes between the two groups.

## Methods

### Ethics statement

This retrospective study received approval from the Institutional Review Board (IRB) of the Chi Mei Medical Center, Taiwan (IRB no. 10705-E04). Before analysis, all personal identifying information was deleted from our dataset with strict anonymity. Consent was waived in the IRB approval.

### Data sources

In March 1995, the Taiwan Department of Health stated the National Health Insurance (NHI) program, which is a required single-payer health care plan with nationwide coverage. The NHI covers approximately 99% of the total Taiwanese population of 23 million and includes 97% of the healthcare providers [10]. The NHI Research Database (NHIRD), managed by the National Health Research Institutes, was developed for research purposes. The NHIRD, one of the largest-scale administrative health care databases in the world, contains all the inpatient and outpatient registration and claim data of the NHI program. It includes patients’ demographic characteristics, disease-diagnostic and surgery-operation codes (based on the International Classification of Diseases, Ninth Revision, Clinical Modification [ICD-9-CM]), prescription data, and hospital costs. In this study, we analyzed the longitudinally linked NHIRD dataset, which consists of a cohort of 1000,000 randomly selected enrollees followed retrospectively from 1996 to 2010. The personal information in the dataset was deidentified with no statistically significant differences in age, sex, and health care cost distributions among the selected subjects.

### Patients

**Fig. 1:**
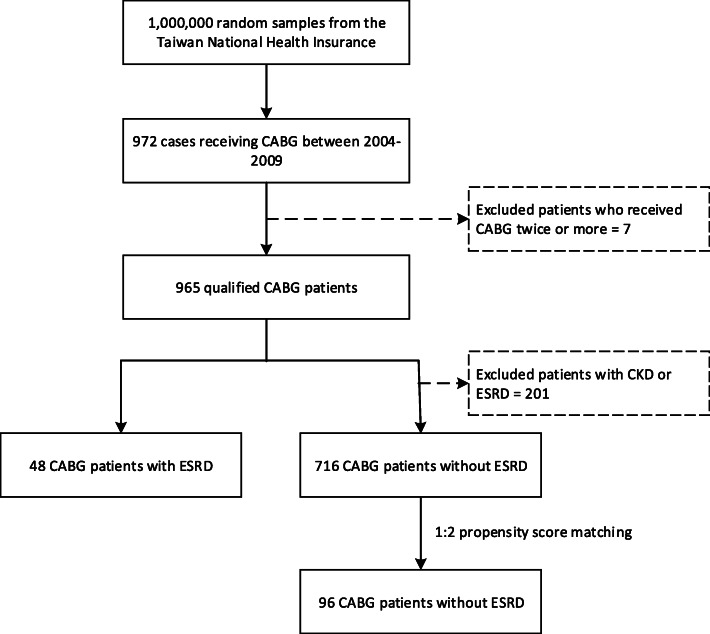
Flowchart of patients selected in the study

This population-based study retrospectively analyzed patients who underwent CABG surgery between January 1, 2004, and December 31, 2009, by identifying records in the NHIRD database in which ICD-9-CM codes 36.1x (bypass anastomosis for heart revascularization) and 36.2x (heart revascularization by arterial implant) were listed as the major operation. In total, 972 qualified cases of patients who had undergone CABG surgery were preliminarily retrieved (Fig. 1). During the study period, only subjects who had a first-time CABG registration were selected to ensure the independence of observations. Cases indicating that the patients received CABG twice or more were excluded. After exclusion filtering, a total of 965 patients remained in our study. The admission date for the CABG operation was designated as the index date. To analyze the complications of CABG patients with an ESRD history, the patients were separated into ESRD and non-ESRD groups by linking them to the Catastrophic Illness Patient Database to obtain the diagnosis of ESRD (ICD-9-CM codes 585 and 586). ESRD patients must register in the catastrophic illnesses database prior to the CABG surgery. After one nephrologist diagnoses ESRD and another confirms it, a catastrophic illness certificate is issued by the Ministry of Health and Welfare’s Health Promotion Administration. ESRD patients receive their catastrophic illness certificates and are exempted from any copayment for hemodialysis and treatment. Thus, the possibility of ESRD identification being a true positive is considered very high.

### Propensity score matching

For each ESRD patient, matched controls were assigned using the propensity score matching method to consider baseline differences between ESRD and non-ESRD patients. The propensity score was estimated from a probability function based on a multivariable logistic regression model to reduce selection bias and balance covariates between ESRD and non-ESRD groups [11]. Covariates included age, sex, hypertension, diabetes, myocardial infarction, stroke, congestive heart failure, peripheral vascular disease, and chronic obstructive pulmonary disease (COPD). The control subjects were matched and selected by using the propensity score ± 0.05 SD at a 2:1 ratio.

### Confounders and outcomes

Patient characteristics and comorbidities were considered confounders in our study. Characteristics included age and sex. Each patient was traced back to 2 years prior to the index date and examined for comorbidities including hypertension, diabetes, myocardial infarction, stroke, congestive heart failure, peripheral vascular disease, and COPD. Mortality and hospital cost outcomes were measured in this study. Mortality outcomes included death during hospitalization and death during postsurgery follow-up. Hospital cost outcomes included intensive care unit (ICU) use (days), ICU cost, ventilator use (days), ventilator cost, length of stay (LOS), and surgical hospital costs for CABG surgery.

In the NHIRD, each hospitalization record contains inpatient information such as in-and-out dates, type of hospitalization (including ICU), operation or surgery codes, and type of transition. The type of transition indicates the posthospitalization states, including discharge with outpatient follow-up required, discharge without outpatient follow-up required, discharge at the acute terminal stage, death during hospitalization, continued hospitalization and transfer. It is worth mentioning that in Taiwan, dying patients prefer to stay home and die there in comfort. Patients discharged at the acute terminal stage were considered to have died during hospitalization (perioperative mortality). Death during postsurgical follow-up (postoperative mortality) included death after discharge from the CABG hospitalization and disenrollment from the NHI. As mentioned above, NHI is a required single-payer insurance program with nationwide coverage. The reasons for disenrollment are death, being missing more than 6 months and emigration. We believe that in our case, patients had disenrollment records because of death. Each hospitalization is also linked to all medical order records with associated expenditures. The ICU costs include physician fees, nurse or caregiver fees, ward fees, medical material consumption, medicines, respirators, nasogastric tube irrigation and meals. All medical costs presented in the study were expressed as New Taiwan Dollars (NTD) with an average exchange rate of 32.50:1 to US dollars during 2004–2009.

### Statistical analysis

Continuous variables were analyzed using Student’s t-test, and categorical variables were analyzed using chi-square tests for the confounders and outcome variables among the ESRD and non-ESRD groups. The absolute standardized difference was used to evaluate the balance between ESRD and matched non-ESRD subjects. Values less than 10% were considered to indicate successful propensity matching [12]. A two-sided *P* value of < 0.05 was considered to indicate statistical significance. Univariate analysis was performed using the Kaplan–Meier estimator with significance determined by the log-rank test to compare the survival function of mortality outcomes for ESRD patients who received CABG. To investigate the main effect of ESRD associated with mortality outcomes, a Cox proportional hazards regression analysis was used with control of the risk factors (sex and age) and 95% confidence intervals (CIs) to estimate the hazard ratios (HRs). Two covariates, age and sex, were used as controlling variables to consider the joint effects in every Cox regression model. Cumulative incidence curves for comparing the mortality rates over time among the ESRD and non-ESRD patients were generated, and KM estimates were used to test for differences between the study groups. All statistical analyses were run in SPSS (version 15, SPSS, Inc., Chicago, IL, USA).

## Results

### Patient characteristics

A total of 965 eligible patients who underwent CABG surgery were identified from the population (Fig. 1). After performing the propensity score matching technique, the study group comprised 48 patients who had a history of ESRD, and the control group had 91 patients without ESRD. The comparisons of basic characteristics between those two groups (ESRD vs. non-ESRD) for patients who received CABG are shown in Table 1. The variables of age, sex and comorbidities were evenly distributed among the two groups such that no significant group difference was found, thereby increasing the between-group comparability.

**Table 1:** Demographics and expenditures of patients with/without ESRD who received CABG

	ESRD	Non-ESRD	*P* value	Absolute standardized difference
	*N* = 48	*N* = 96		
Age (mean ± SD)	62.04 ± 10.04	61.63 ± 11.77	.838	0.0375
≦39 (%)	1 (2.1)	3 (3.1)	.954	
40–49	6 (12.5)	10 (10.4)		
50–59	15 (31.3)	29 (30.2)		
60–69	12 (25.0)	29 (30.2)		
≧70	14 (29.2)	25 (26.0)		
Sex (%)				
Male	32 (66.7)	66 (68.8)	.800	0.0449
Female	16 (33.3)	30 (31.3)		
Comorbidities (%)				
Hypertension	34 (70.8)	62 (64.6)	.453	0.1328
Diabetes	32 (66.7)	69 (71.9)	.520	0.1129
Myocardial infarction	14 (29.2)	31 (32.3)	.703	0.0672
Stroke	15 (31.3)	21 (21.9)	.221	0.2139
Congestive heart failure	28 (58.3)	52 (54.2)	.635	0.0827
Peripheral arterial disease	10 (20.8)	14 (14.6)	.343	0.1629
COPD	8 (16.7)	18 (18.8)	.759	0.0549
Expenditure in CABG (mean ± SD)				
ICU use (day)	11.06 ± 12.18	7.24 ± 8.89	.058	
ICU cost (1000NTD)	28.75 ± 31.80	17.99 ± 21.68	.030	
Ventilator use (day)	10.21 ± 12.34	6.40 ± 11.05	.064	
Ventilator cost (1000NTD)	15.99 ± 20.09	10.06 ± 17.73	.074	
Length of stay (day)	21.47 ± 12.69	17.71 ± 10.07	.094	
Surgery cost (1000NTD)	565.20 ± 300.31	421.89 ± 247.74	.010	
Mortality (%)				
Perioperative	5 (10.4)	2 (2.1)	.028	
Postoperative	16 (33.3)	11 (11.5)	.002	
Follow-up years (median)	3.10	4.06		

### Mortality among ESRD and non-ESRD

As shown in Table 1, patients in the ESRD group had marginally higher ICU stays (11.06 vs 7.24 days) and significantly higher ICU costs (28,750 vs 17,990 NTD) than those in the non-ESRD group. Similarly, patients in the ESRD group had significantly higher surgery costs (565,200 vs. 421,890 NTD), a higher perioperative mortality proportion (10.4% vs 2.1%) and a higher mortality proportion (33.3% vs 11.5%) during the postsurgical follow-up than those in the non-ESRD group. Ventilator use, ventilator cost and LOS were trending higher in the ESRD group but were not significant. In Table 2, the adjusted HR (3.55; 95% CI 1.64–7.69; *P* = 0.001) indicated that the mortality risk was 3.55-fold higher for the ESRD patients than for the non-ESRD patients. This relationship was further investigated to examine the association with comorbidities. For each comorbidity variable, two Cox regression models were used to investigate the mortality for patients with or without comorbid disease. The Cox regression showed that those comorbidities were associated with higher mortality risks in the ESRD patients than in the non-ESRD patients. For example, for the patients who had stroke, the adjusted HR of mortality in the ESRD patients was 7.15 (95% CI 1.45–35.23; *P* = 0.016) times that of the non-ESRD patients. For the patients who did not have stroke, the adjusted HR was 2.78 (95% CI 1.07–7.21; *P* = 0.035).

**Table 2:** Mortality in patients with and without ESRD who received CABG

	ESRD (*N* = 48)	Non-ESRD (*N* = 96)	Adjusted HR (95% CI)	*P* value
Mortality (%)	16 (33.33)	11 (11.46)	3.55 (1.64–7.69)	.001
Mortality with comorbidities (%)				
Hypertension				
Yes	12 (25.00)	10 (10.42)	2.69 (1.16–6.26)	.022
No	4 (8.33)	1 (1.04)	11.25 (1.25–101.16)	.031
Diabetes				
Yes	13 (27.08)	9 (9.38)	4.32 (1.78–10.47)	.001
No	3 (6.25)	2 (2.08)	2.93 (0.49–17.57)	.240
Myocardial infarction				
Yes	8 (16.67)	6 (6.25)	3.68 (1.28–10.64)	.016
No	8 (16.67)	6 (6.25)	3.64 (1.19–11.17)	.024
Stroke				
Yes	7 (14.58)	3 (3.13)	7.15 (1.45–5.23)	.016
No	9 (18.75)	8 (8.33)	2.78 (1.07–7.21)	.035
Congestive heart failure				
Yes	9 (18.75)	9 (9.38)	2.26 (0.87–5.87)	.093
No	7 (14.58)	2 (2.08)	10.11 (2.09–48.99)	.004
Peripheral arterial disease				
Yes	4 (8.33)	2 (2.08)	3.20 (0.59–17.51)	.179
No	12 (25.00)	9 (9.38)	3.43 (1.44–8.17)	.005
COPD				
Yes	5 (10.42)	3 (3.13)	5.50 (1.29–23.53)	.021
No	11 (22.92)	8 (8.33)	3.18 (1.28–7.93)	.013

For each comorbidity variable, two Cox regression models were conduct to investigate the mortality for patients having or not having the comorbid disease

### Mortality with other risk factors

The joint effects of mortality between ESRD and associated risk factors were further investigated. As shown in Table 3, the ESRD patients with a history of myocardial infarction had a significantly higher mortality risk (aHR 2.83; 95% CI 1.06–7.58; *P* < 0.05), indicating that the risk of death for ESRD patients with myocardial infarction history was 2.83-fold higher than that for those without myocardial infarction. Additional interaction effects between ESRD (with/without) and the risk factors (with/without) were also assessed (Table 4). The interaction terms were significant in Diabetes × ESRD (aHR 4.19; 95% CI 1.94–9.07; *P* < 0.01) and Myocardial infarction × ESRD (aHR 4.86; 95% CI 2.11–11.16; *P* < 0.01).

**Table 3:** Mortality in ESRD patients who received CABG

Variable	Event (%)	Death	Proportion	aHR (95% CI)	*P* value
Total	48	16	0.33		
Hypertension	34 (70.83)	12	0.35	1.31 (0.42–4.06)	.644
Diabetes	32 (66.67)	13	0.41	2.32 (0.66–8.17)	.189
Myocardial infarction	14 (29.17)	8	0.57	2.83 (1.06–7.58)	.038
Stroke	15 (31.25)	7	0.47	2.31 (0.85–6.26)	.099
Congestive heart failure	28 (58.33)	9	0.32	0.87 (0.32–2.33)	.780
Peripheral arterial disease	10 (20.83)	4	0.40	1.46 (0.47–4.54)	.511
COPD	8 (16.67)	5	0.63	2.43 (0.84–7.02)	.100

**Table 4:** Interaction between ESRD and comorbidities for patients received CABG in Mortality

Interaction	aHR (95% CI)
Hypertension × ESRD	0.24 (0.02–2.54)
Diabetes × ESRD	4.19 (1.94–9.07)*
Myocardial infarction × ESRD	4.86 (2.11–11.16)*
Stroke × ESRD	2.32 (0.86–6.27)
Congestive heart failure × ESRD	0.22 (0.04–1.35)
Peripheral arterial disease × ESRD	1.16 (0.17–7.83)
COPD × ESRD	1.71 (0.31–9.38)

*P-value < 0.01

### Cumulative incidence functions of mortality

**Fig. 2:**
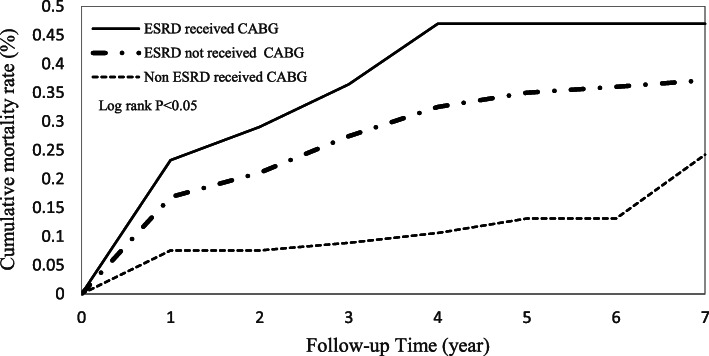
Cumulative mortality rate in patients with and without ESRD who received CABG

**Fig. 3:**
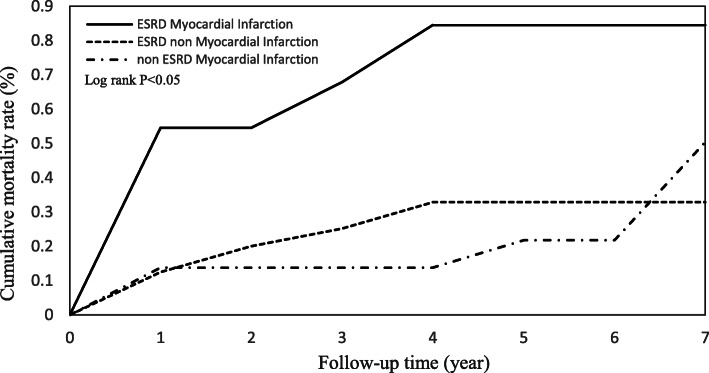
Cumulative mortality rate for ESRD patients with myocardial infarction history

Cumulative incidence functions for the ESRD and non-ESRD patients illustrated the mortality rates over time (Fig. 2). There were significant differences between the two study groups after patients received CABG surgery. Figure 3 shows that ESRD patients with a myocardial infarction history had the highest mortality risk compared to ESRD patients with no myocardial infarction history and non-ESRD patients with a myocardial infarction history.

## Discussion

Our study differs from previous studies that compared CABG and PCI in ESRD patients because we performed propensity score matching from a nationwide database before comparing ESRD patients who received CABG with patients without ESRD who received CABG. Our study controlled for confounding variables by propensity score matching and tried to delineate the differences between these two groups and calculate their medical costs. There were some noteworthy findings in our study. First, ESRD patients had a higher perioperative and postoperative mortality than non-ESRD patients after CABG. There was also a significant difference in ICU costs and surgical costs. However, the difference in duration of ICU stay did not approach statistical significance (*P* = 0.058). Second, the risk factors for mortality after CABG were hypertension, diabetes, myocardial infarction history, stroke and COPD in ESRD patients compared with non-ESRD patients. Third, among ESRD patients after CABG, myocardial infarction was the only risk factor for mortality.

### Mortality in ESRD and non-ESRD after CABG

Powell et al. [9] compared the mortality after CABG in ESRD patients on dialysis (*N* = 28) and in patients with normal renal function (*N* = 84) in a single hospital. In their study, there were no significant differences in perioperative morbidity or mortality in either group after CABG. However, there were only 2 patients who died in the ESRD group and 1 patient who died in the non-ESRD group. The ESRD cases were matched to non-ESRD (1:3) on the basis of age, gender, smoking history, and New York Heart Association Functional Class. Moreover, there was a significant difference in comorbidities between ESRD and non-ESRD patients, including hypertension, diabetes, pulmonary hypertension, and vascular disease. We matched covariates including age, sex, hypertension, diabetes, myocardial infarction history, stroke, congestive heart failure, peripheral vascular disease, and COPD. In our study, we found that ESRD patients had a higher mortality after CABG compared with patients with normal renal function not only during hospitalization but also during follow-up for 7 years. Even after controlling for multiple comorbidities, the prognosis of ESRD patients remained worse than that of non-ESRD patients. Other risk factors, such as inflammation, are increasingly apparent with atherosclerosis in hemodialysis patients and result in the development of cardiovascular disease as well as morbidity and mortality [13]. Indeed, there is growing evidence that proinflammatory cytokines, such as IL-1, IL-6, TNF-α and CRP, contribute to atherosclerosis and adverse cardiovascular outcomes and predict mortality in ESRD patients [14–17].

### Hospital costs for ESRD and non-ESRD patients after CABG

Powell et al. [9] found no significant difference between ESRD patients and non-ESRD patients in mortality, ICU LOS and total length of hospitalization after the multivariate analysis. Our study found that mortality and medical cost were significantly different between the two groups. Although not significant, our ESRD patients also tended to have increased ICU days, ventilator days, ventilator cost and length of hospital stay after CABG compared with those in patients without ESRD. Charytan et al. [18] used ICD-9-CM codes to analyze administrative data for over seven million patients and enrolled 635 ESRD patients who underwent CABG. The average length of hospital stay was 15.7 days in ESRD patients and 9.6 days in non-ESRD patients. The average medical cost was 104,347 U.S. dollars for each ESRD patient and 67,271 U.S. dollars for each non-ESRD patient. The NHI is a public program run by the government based on single-payer, universal, and compulsory policies. Life expectancy in Taiwan has subsequently increased, but healthcare costs are far lower in Taiwan than in most highly developed countries in Europe and North America, that is, at US $1430 per capita per year, representing just 6.3% of the Taiwanese GDP in 2016.

### Prognostic factors among ESRD patients after CABG

Yamauchi et al. compared data from the Japan Adult Cardiovascular Surgery Database on 1300 ESRD patients and 18,387 non-ESRD patients who received CABG. ESRD patients had a higher 30-day mortality rate and operative mortality than non-ESRD patients. Another study [19] enrolled 279 dialysis-dependent renal failure patients and showed that these patients were 3.1 times more likely to die after CABG after adjusting for factors of age, gender, previous heart operation, left ventricular ejection fraction, and severity of stenosis. Our study also showed increased mortality in ESRD patients with an adjusted HR of 3.55 (95% CI 1.64 to 7.69; *P* < 0.001). Sezai et al. presented the risk factors for postoperative mortality, including acute myocardial infarction, advanced age, preoperative intra-aortic balloon pumping and concomitant surgery [20]. Gelsomino et al. [21] retrospectively analyzed 28 ESRD patients undergoing cardiac surgery and found that poor left ventricle function and Canadian Cardiovascular Society (CCS)/New York Heart Association (NYHA) functional class IV were related to early and late mortality. Because of the limitations of the database, we lack information on echocardiographic reports and the NYHA functional class to classify congestive heart failure patients based on their symptoms.

Different risk factors were found to be associated with mortality among ESRD patients who received CABG. Tong et al. [22] analyzed mortality risk factors in Asian ESRD patients and reported that in ESRD patients with cardiovascular disease, stroke was a significant risk factor in all-cause mortality, while diabetes and hs-CRP were significant risk factors in all-cause mortality in ESRD patients without cardiovascular disease. ESRD patients with regular hemodialysis, old age, diabetes, and hypertension are at increased risk of stroke [23–25], but our study found that these factors were not significantly associated with mortality among ESRD patients after CABG. In our study, myocardial infarction history was an independent predictor of all-cause mortality after CABG in ESRD patients. Emergency CABG was performed for impending or acute myocardial infarction, but the optimal timing of CABG after myocardial infarction remains the subject of various discussions. A previous history of myocardial infarction may lead to myocardial tissue death, which is not reversible after CABG.

### Limitations

Our study has limitations, and due to the small number of events (only 27 deaths), there is limited power to detect risk factors for mortality and make comparisons between ESRD and non-ESRD. Therefore, these findings should be cautiously interpreted. The limitations of our study are the common limitations of other administrative datasets. Moreover, the present investigation lacks essential laboratory data as well as data from modalities such as cardiac imaging, echocardiography and angiography; thus, we cannot quantify the ejection fraction of the left ventricle and cannot assess the severity and location of coronary artery stenosis.

In addition, our study contains little information on socioeconomic status, lifestyle, healthy behavior, or unhealthy habits. Otherwise, researchers may doubt the accuracy of disease coding. The diagnostic codes in the database are principally meant for billing purposes and thus may not be accurate enough by themselves. However, there is a way to overcome this limitation. Our ESRD patients were screened from the Registry for Catastrophic Illness Patient Database, which secures reliable identification. Entry into this registry, which waives copayment of the enrollee, is permitted only after explicit criteria and another physician examination are fulfilled. The primary diagnoses for a severe illness, such as ischemic stroke or acute myocardial infarction, have been validated and are also convincing in general.

## Conclusions

Compared with non-ESRD patients, ESRD patients who received CABG had higher mortality during hospitalization and after long-term follow-up. The hospital costs and utilization of health care were also higher in ESRD patients after CABG. Improving ESRD patient care after CABG has become a priority for health care providers.

## Data Availability

Data are available from the National Health Insurance Research Database (NHIRD) published by the Taiwan National Health Insurance (NHI) Bureau. Due to legal restrictions imposed by the government of Taiwan in relation to the “Personal Information Protection Act”, data cannot be made publicly available.

## Abbreviations

aHR: adjusted hazard ratios
CABG: Coronary artery bypass graft
COPD: Chronic obstructive pulmonary disease
CKD: Chronic kidney disease
ESRD: End-stage renal disease
HR: Hazard ratios
ICD-9-CM: International Classification of Diseases, Ninth Revision, Clinical Modification
ICU: Intensive care unit
LOS: Length of stay
NHI: National Health Insurance
NHIRD: National Health Insurance Research Database
PCI: Percutaneous coronary intervention
